# Current management of cancer pain in Italy: Expert opinion paper

**DOI:** 10.1515/med-2021-0393

**Published:** 2021-12-06

**Authors:** Franco Marinangeli, Annalisa Saetta, Antonio Lugini

**Affiliations:** Department of Anesthesiology Intensive Care and Pain Treatment, University of L’Aquila, Località Coppito, Piazzale Salvatore Tommasi, 1-67100, L’Aquila, Italy; Department of Oncology and Hematology, Humanitas Clinical and Research Center, 20089 Rozzano (Milan), Italy; Department of Oncology, San Giovanni-Addolorata Hospital, 00184, Rome, Italy

**Keywords:** cancer pain, breakthrough cancer pain, multidisciplinary team, rapid-onset opioids, fentanyl

## Abstract

**Introduction:**

Chronic pain and breakthrough cancer pain (BTcP) have a high prevalence in all cancer types and cancer stages, combined with a significant physical, psychological, and economic burden. Despite efforts to improve appropriate management of cancer pain, a poor assessment and guilty undertreatment are still reported in many countries. The purpose of this expert opinion paper is to contribute to reduce and clarify these issues with a multidisciplinary perspective in order to share virtuous paths of care.

**Methods:**

Common questions about cancer pain assessment and treatment were submitted to a multidisciplinary pool of Italian clinicians and the results were subsequently discussed and compared with the findings of the published literature.

**Conclusion:**

Despite a dedicated law in Italy and effective treatments available, a low percentage of specialists assess pain and BTcP, defining the intensity with validated tools. Moreover, in accordance with the findings of the literature in many countries, the undertreatment of cancer pain is still prevalent. A multidisciplinary approach, more training programs for clinicians, personalised therapy drug formulations, and virtuous care pathways will be essential to improve cancer pain management.

## 1 Introduction

In the systematic review and meta-analysis by van den Beuken-van et al., in 2016, pain prevalence rates were 50.7% in all cancer stages; prevalence rates were 39.3% after curative treatment, 55.0% during anticancer treatment; 66.4% in advanced, metastatic, or terminal disease [1]. A high prevalence of pain has been documented in haematology patients at the time of diagnosis, during therapy, and in the last month of life [2].

Chronic pain is also present in about half of the cancer survivors [3,4], and approximately 5–20% of these people complain of severe pain that interferes with quality of life.

Despite improved pain assessment in clinical practice, patient awareness, and effective treatments, cancer-pain may be inadequately controlled approximately in up to one-third of the patients [5,6], especially in elderly patients [7]. Pain severity resulted in 38.0% prevalence of moderate to severe pain, from 31% after curative treatment to 45% in advanced cancer [1,8]; unfortunately, severe pain management is often inadequate, even for people facing the end-of-life transition with palliative and hospice care [9,10].

With reference to types of cancer, pain has a high prevalence earlier in pancreatic cancer [11], head and neck cancer, lung and breast cancer [1], and non-Hodgkin’s lymphoma [11].

A high percentage of cancer patients, ranging from 33 to 95% in different studies, also suffer from breakthrough cancer pain (BTcP), two to three times a day [12,13,14]. BTcP is a “transient exacerbation of pain that occurs despite relatively stable and adequately controlled background chronic pain” [15].

BTcP varies according to the stage of the tumour, with >70% in the advanced stages [16], and to the type of the tumour, with the highest prevalence in patients with pancreatic cancer (71%) and colorectal cancer (62%), and the lowest prevalence among patients with multiple myeloma (32%) and lymphoma (22%) [17].

High intensity of BTcP was described by 75.5% of the patients [14], and the painful crises can be exacerbated by either predictable or unpredictable events [12,15,18], but always associated with a significant physical, psychological, and economic burden [19,20]. Anxiety, depression, and sleep disorders are common consequences, and the inevitability of further episodes generates fear in many patients, with a consequent reduction in daily activities, mobility, family, and social relationships [19,21].

If cancer pain may be inadequately controlled, specific types of pain, such as BTcP, is more likely to be underestimated and undertreated [17]; clinical inertia is still a serious problem in the handling of BTcP [22].

It is therefore essential to carry out an individualised and very comprehensive initial assessment to provide the best management approach in patients with cancer pain; cancer pain treatment has to comply with the guideline’s recommendations, taking advantage of modern technologies in drug formulations, and always considering the tailoring to the patient, with careful monitoring of pain relief.

## 2 Materials and methods

This study is an expert opinion approach in the evaluation of the management of cancer pain by Italian clinicians and comparing the results with the recommendations of the literature.

The scientific board developed seven main questions for use in cancer pain assessment and decision analysis to treat; the questions were submitted as anonymous branching survey that utilises skip patterns to ensure that respondents are asked only those questions that apply to them, guiding respondents based on their profile. The invitation to the survey declared the content of the study and gave the clinicians an opportunity to join the project or deny the involvement; all the clinicians have indicated their willingness to voluntarily take part in the study. Ethics committee approval is not required for this anonymous survey.

The branching survey focusing on the approach to chronic cancer pain and BTcP was submitted to 13 oncologists (41%), 9 algologists (28%), and 10 palliativists (31%) from different Italian regions and institutions and from different care settings: oncology departments, pain centres, hospice, and palliative home care. The survey questions are detailed in Table 1.

**Table 1:** Survey questions

In your workplace, what is the path used in the assessment of pain in cancer patient?
In the management of cancer pain, which drug do you use mainly?
In the management of cancer pain, when do you prefer the transdermal route?
How do you recognize episodes of BTcP?
Which characteristic of a rapid-onset opioid is the most important feature of your choice?
Do you believe it is an advantage to use the same molecule (fentanyl) to control basic pain and episodes of BTcP?
Do you think it is an advantage to have a ROO with different dosages compared to other drugs on the market?

Participants declared to visit more than 20 patients per month (53%) or 15–20 patients per month (38%) with chronic cancer pain, and from 10 to more than 20 patients per month (34%) or from 5 to 10 patients per month (66%) with BTcP. Ninety-two percent believe they have adequate training to easily recognise the BTcP and only 8% would like better training.

The results of the survey were subsequently discussed together by the participants in two mixed focus group meetings held in October and November 2020, to ensure a multidisciplinary view of cancer pain management.

The meeting was recorded by Sanitanova S.R.L. and a medical writer provided a meeting report to all the participants for approval of the contents and to allow the authors to proceed to the comparison with the data of the literature.

For resource supporting the search and retrieval of medical literature, the authors opted for the PubMed database using the keyword “cancer pain” in the title of the article; the research has been restricted to the period 2009–2019, because most of the articles on cancer pain were published only in this decade; previous publications were left only if particularly significant on the topics. Among the 1,728 articles in English language only clinical trials, meta-analysis, randomised control trials, and reviews or systematic reviews were taking into consideration. More keywords (“assessment,” “breakthrough,” and “breakthrough AND opioids”) have been added to further narrow the bibliographical research in the different chapters. A further selection was made on the best matches or based on the journal impact factor or notoriety of the authors on the topic “cancer pain”


**Ethics statement:** The conducted study is not related to either human or animals use. Ethical committee approval is not required for this anonymous survey study.

## 3 Discussion

### 3.1 An important consideration for adequate pain control in patients with cancer is the early, appropriate, and regular assessment of pain

Pain assessment tools should be used routinely in monitoring patients with cancer, beginning in the early stages of cancer, always over the course of treatment and certainly in cancer survivors [23,24].

Since pain is a subjective perception, patients should be actively involved in the evaluation process and individual characteristics including age, cognitive function, psychological aspects, patient’s ability to cope, and all components of distress or suffering must be considered during pain assessment [25,26,27,28,29].

Intensity is one of the most relevant standards for pain assessment. The intensity of pain and the treatment outcomes should be assessed regularly and consistently using the visual analogue scale (VAS) or the numerical rating scales (NRS). In elderly patients, or when cognitive deficits are severe, observational scales are alternative strategy for assessing the presence of pain [30,31]. Pain localisation is an integral part of pain assessment. Cancer patients often have pain in more than one site and it is necessary to include a body map in pain assessment tool to precisely define the pain areas. Body map drawings are included in many assessment tools like the McGill Pain Questionnaire or the Brief Pain Inventory. Computerised methods to analyse body map pain are being developed and may aid to measure the pain area more precisely and specify pain intensity scale for each pain location, improving communication between patients and healthcare and between healthcare providers [32,33,34].

In palliative care setting or in dying patients, pain intensity is moderate to severe [35,36] and is often referred to many sites [37], commonly reported as having complex qualities and susceptible to variations in its pattern [38,39]. At the very end of life, it could be often harder for the patient to report pain and its location or characteristics. So it is important to use behavioural assessment tools for persons who are not able to communicate their pain [40].

In Italy, in order to guarantee pain care, a proper law (Law 38/2010, Measures to Guarantee the Access to Palliative and Pain Treatments) was enacted in 2010 [41]. The law mandates doctors and nurses to do a pain assessment at fixed intervals and to enter the data in their medical records. Unfortunately, this obligation is often disregarded.

In our survey, only 26% of respondents expect that pain is a dimension to be investigated both during hospitalisation and at any outpatient access, and to be measured every day by the doctor or the nurse (17%). In 22% of the cases, voters believe that on the first visit the patient is encouraged to report the appearance of pain during care; only if the pain is reported by the patient or if the specialist believes that there is evidence to suspect it, then it is appropriate for a specific assessment. Only 13% of the oncologists and palliativists require the expertise of a pain specialist for pain assessment.

Despite background pain being adequately controlled, patients frequently experience episodes of acute pain exacerbation known as BTcP. Diagnosis of BTcP needs a well-controlled basal pain with a therapy round the clock, able to provide analgesia to a mild pain intensity for most hours of the day. At the end of life, BTcP becomes an even worse distressing issue with a daily occurrence and high intensity involving almost all cancer patients [16,42]. Four to seven episodes per day may occur [11]. Its impact is also important in the early stages of cancer, so patients need to be assessed accurately at any stage [18].

The lack of validation of BTcP tools has been a limitation; however, some assessment tools for BTcP have been successfully used, from the first Breakthrough Pain Questionnaire (BPQ) in 1990 [12,43] to the Alberta Breakthrough Pain Assessment Tool, ABPAT, and the Pain Assessment Tool (BAT) [44,45].

**Figure 1:**
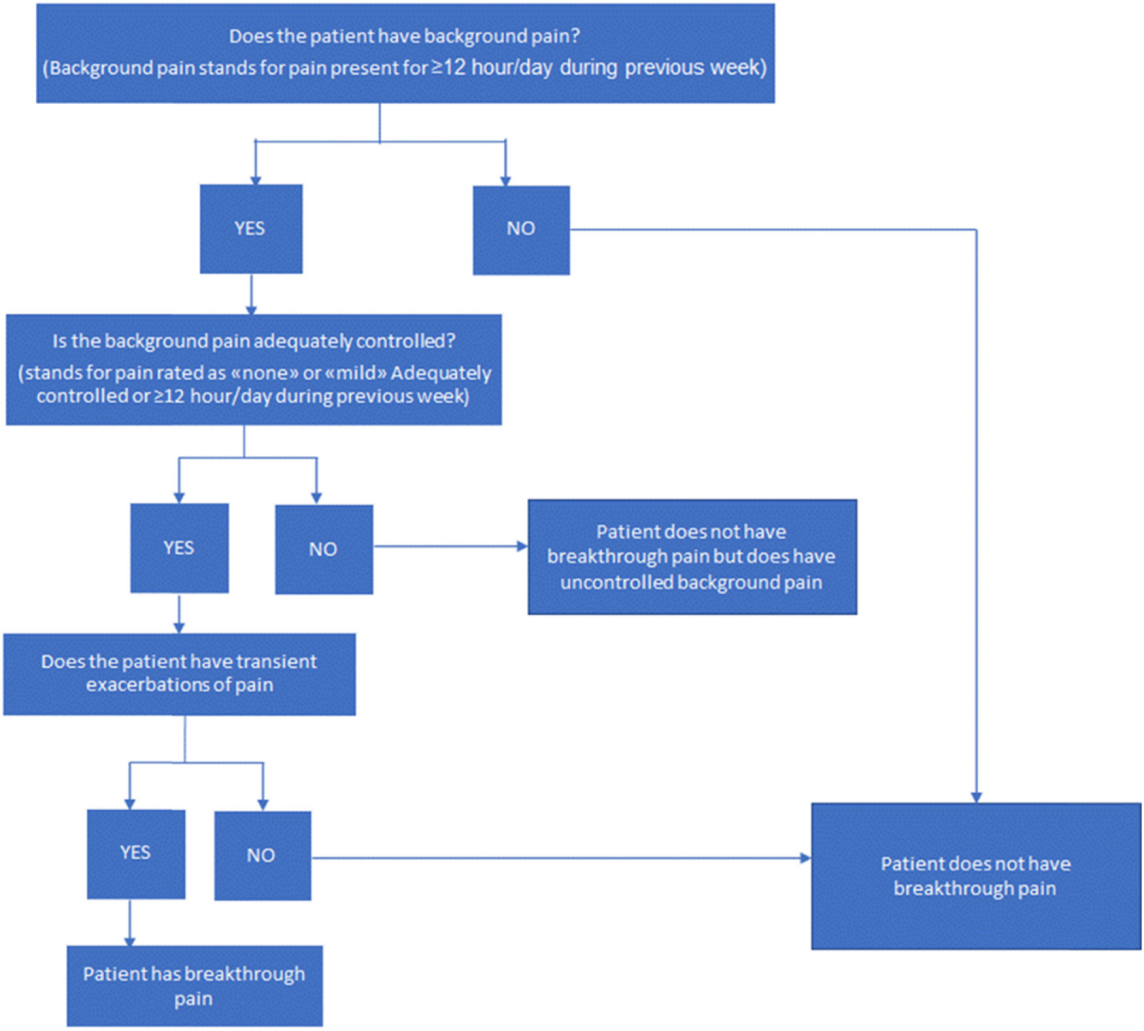
Davies algorithm [15].

The Edmonton Classification System for Cancer Pain is a classification tool that evaluates different dimensions of pain and investigates the existence of factors that could add clinical complexity [46], even though the simple three-questions algorithm proposed by Davies et al. [15] is widely used and cited in the literature (Figure 1).

Camps Herrero et al. reported a prevalence of 91% of BTcP using the Davies algorithm [22] and in a 2019 Expert consensus, participants agreed (75.8%) in the application of the Davies algorithm for diagnosis of BTcP in older patients [47]. Sixty-five percent of the experts surveyed stated that they actively assess the presence of BTcP and define their intensity using the NRS scale. Thirty-one percent report that the patient (or caregiver) spontaneously relates episodes of intense pain with the characteristics of BTcP.

### 3.2 Pain reduction and improvement in quality of life are the most important treatment goals in cancer patients; cancer pain treatment must be tailored

The management of cancer-related pain is based on three steps of the WHO analgesic ladder, according to pain intensity [48,49]; however, pain therapy must also be based on the mechanisms underlying the onset of pain [50,51]. Many patients with cancer pain experience a *mixed pain syndrome* with a substantial overlap of nociceptive and neuropathic symptoms. The combination of different pain is not only directly linked to the tumour (by compression or infiltration) or due to metastasis or cancer therapies [52,53] but even to other aetiologies, unrelated to cancer [8,54]. Also, in the 4 months prior to death, pain seems to be linked to nociceptive or neuropathic mechanisms or both [55,56].

Strong opioids are the mainstay of analgesic therapy in treating cancer-related pain. WHO cancer pain ladder recommends, for mild to moderate pain, weak opioids such as tramadol and codeine in combination with non-opioid analgesics, but low doses of strong opioids could be an option as an alternative to weak opioids [31,57,58].

The guidelines [31,57,59] recommend prescribing the lowest initial dose of immediate release opioids to patients who are started with opioids; oxycodone or hydromorphone, both have immediate-release and modified-release formulations for oral administration, are effective alternatives to oral morphine in moderate to severe pain [60], and the combination of oxycodone/naloxone could reduce opioid-induced constipation [61]. If the patient is expected to need long-term treatment, extended-release opioids at equianalgesic doses may be chosen. Fentanyl transdermal administration is indicated for the treatment of severe chronic pain that requires continuous administration of long term opioid, and it is best recommended for patients in stable treatment with opioids [31]. Due to its high lipid‑solubility, low molecular weight, and good skin absorption effects, fentanyl is suitable to use transdermally, as a patch [62,63], allowing to eliminate gastrointestinal absorption and first-pass metabolism; thus, lower drug dosages can be used, reducing the incidence of adverse effects and avoiding gastric irritation [64].

Transdermal fentanyl can be useful in opioid-naïve patients with nausea, vomiting, and problems with swallowing and is usually a good option in head and neck cancers, due to safety and tolerability profiles to control baseline pain [65]. Fentanyl and buprenorphine are indeed the safest opioids in patients with chronic kidney disease stage 4 or 5 (estimated glomerular filtration rate < 30 mL/min) [31,66].

Thanks to new technologies, the transdermal drug delivery systems are designed to ensure increasingly more simple and non-invasive methods of drug delivery and provide additional benefits for patients, enhancing their compliance. Technology of transdermal system can further improve the effectiveness of the device. A modern and innovative transdermal fentanyl five layers patch contains a rate-controlling membrane designed to control a constant release of drug over 72 h. Fifteen percent of the drug loaded in the adhesive layer immediately in contact with the skin allows to overcome the long delay that precedes the onset of the drug in the plasma after the first administration and good adhesion properties to the skin, avoiding that the patch comes off ahead of time [67].

Patients with specific conditions that prevented the administration of oral opioids (especially difficulties in swallowing, nausea, and vomiting) or dying people should be considered to receive intravenous continuous infusion of morphine which will ensure a rapid-onset time for background pain control due to a complete bioavailability. The subcutaneous route can be considered as a safe and valid alternative to the intravenous and transdermal fentanyl may be the better choice to ensure a suitable alternative analgesic therapy for outpatients and for end-of-life home patients [68,69,70,71].

**Figure 2:**
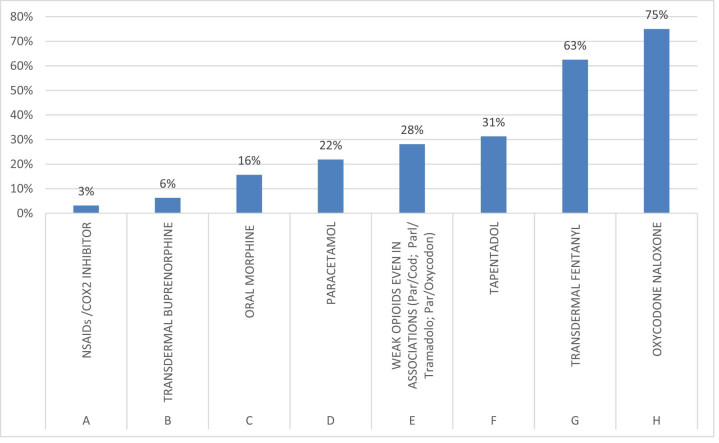
Survey question: “In the management of cancer pain, which drug do you use mainly?.”

The combination of opioid oxycodone/naloxone for oral administration represents the main choice of experts (75%) invited to the survey for the management of cancer patients with pain. Transdermal fentanyl is also widely used by the clinicians surveyed and represents the second most voted option (63%) among multiple choice answers (Figure 2).

In our survey, 19% of the voters prefer transdermal fentanyl in some target patients such as young people or cancer patients under active therapy, with good performance, and work activity, so as not to interfere with their daily activities and reduce perceived medicalisation. Elderly patients with poor renal function are also a target where the transdermal route is preferable.

Fifty-three percent of experts do not use transdermal fentanyl in opioid-naïve patients. Twenty-two percent of clinicians choose the transdermal route in multi-treated patients for comorbidity, to improve compliance by reducing oral administrations.

When dose increases do not provide analgesia due to tolerance development or adverse intolerable and unmanageable side effects appear, chronic pain patients frequently need to be switched to alternative opioid or alternative route of administration [72,73,74,75].

In this survey, 6% of experts chose transdermal fentanyl mainly in opioid switching, also known as opioid rotation, for improving pain relief or reducing the intensity of adverse effects.

### 3.3 Fentanyl is also the best molecule suited to treat BTcP

A recent national, multicentre study [76] involved 4,016 Italian patients with BTcP to identify potential variables influencing the clinical presentation of BTcP; 64.4% of patients had 1–2 episodes per day, 29.4% had 3–4 episodes per day, and 6.2% had 5 episodes of BTcP/day. Patients with head and neck cancer and pancreatic cancer had a higher number of BTcP episodes per day. Bhatnagar et al. also reported a high prevalence of BTcP experienced by patients with head and neck cancer; prevalence is 48% (average of 3.85 episodes per day) of which more than 50% of episodes were of gradual onset but with severe intensity [77].

The most frequent sites of pain are vertebrae (36.7%), abdomen (29.5%), extremities (19.5%), chest wall (18.7%), and pelvis (10%). Pain mechanism was mixed (61.8%), nociceptive (30%), and neuropathic (8.1%) [76].

Time to maximum pain intensity was ≤10 min (fast-onset BTcP) in 68.9% of patients and >10 min (slow-onset BTcP) in 31.1% of patients; those with fast-onset BTcP had a higher number of BTcP. The mean intensity of BTcP was 7.5 (SD 1.3) in 31.1% of patients. The mean duration of untreated BTcP episodes was 43.3 min [76].

Considering the characteristics of BTcP (rapid onset, short duration) it is clear that slow-onset oral drugs cannot provide an adequate pain relief [31,76,78].

Intravenous or subcutaneous morphine injections provide effective and very fast treatment of BTcP episodes without producing relevant adverse effects or adding risks [79,80]. Data from the literature confirmed that intravenous morphine is a valid option for the treatment of BTcP episodes in patients who are already in treatment with intravenous morphine infusion [78,81]. Parenteral opioids may also be administered by the patients, with the use of a patient-controlled analgesia device [82].

However, invasive routes of administration are unpleasant for the patient and intravenous opioids administration require expertise and appropriate setting.

Different fentanyl formulations have been developed to provide, by non-invasive routes, fast pain relief, such as transmucosal fentanyl formulations, called rapid-onset opioids (ROOs), provide a rapid effect after drug administration, with a shorter titration period, significantly lower the impact on quality of life and result in better satisfaction scores [83,84]. As these products have been tested only in opioid-tolerant patients, the current recommendation for use is only for patients receiving doses of oral morphine equivalents of at least 60 mg daily, for a week or longer.

Several placebo-controlled randomized controlled trials have demonstrated the efficacy of all available transmucosal fentanyl formulations for BTcP such as oral, transmucosal buccal tablet, sublingual tablet, buccal soluble film, and sublingual and intranasal spray [85,86,87,88], but there is no evidence of superiority for any specific formulation or delivery systems.

There are at least two important factors that should always be considered when choosing BTcP pharmacological treatments: baseline opioid regimen and breakthrough pain’s characteristics and patient preferences. Furthermore, other clinical conditions such as oral mucositis, integrity alteration of the nasal mucosa, presence of vascular access device, patients’ self-ability and dexterity in taking medication, and patient preference should be assessed for a tailored BTcP pharmacological treatment [89,90,91].

Each formulation of rapid-onset fentanyl, according to the summaries of product characteristics, should be carefully titrated to an optimal maintenance dosage that provides satisfactory analgesia for ongoing treatment of breakthrough pain episodes and acceptable level of adverse effects. Moreover, because of the considerable differences in absorption profiles and bioavailability among various ROOs, switching to other formulation of the dose should always be titrated.

Table 2 shows the main peculiarities of the transmucosal fentanyl products with marketing authorisation in Italy.

**Table 2:** Data concerning Italian transmucosal fentanyl products with marketing authorisation by the Italian Medicines Agency (Giovambattista Zeppetella & Davies, 2015; Italian Medicines Agency 2020 available at http://www.agenziafarmaco.gov.it/. Accessed May 5, 2020)

Data concerning Italian transmucosal fentanyl products with marketing authorisation						
	Vellofent^®^	Effentora^®^	Abstral^®^	Instanyl^®^	Pecfent^®^	Actiq^®^
	Fentanyl citrate sublingual tablets	Fentanyl buccal tablets	Fentanyl sublingual tablets	Fentanyl nasal spray	Fentanyl citrate nasal spray	Fentanyl citrate oral transmucosal lozenge
Route of administration	Sublingual rapid release tablets	Buccal/sublingual effervescent tablets	Fast dissolving sublingual tablets	Intranasal spray solution	PecSys nasal drug delivery system	Compressed lozenge with integral oromucosal applicator
Absolute bioavailability compared to intravenous fentanyl (%)	About 70	65 (±20%)	54	89	60	50
*T*_max_ (maximal concentration time, min)	50–90	47	22,5–240	12–15	15–21	91 (20–480)
Onset of action (min)	6	15	10	10	5–10	20–40

Adverse reactions attributed to BTcP medications are rare and their intensity is mild. There is a strong consensus for pharmacological treatment with transmucosal fentanyl with “start low and go slow” dosage method even for the older population [47]. Indeed, older people are more at risk of developing adverse effects due to the well-known lower tolerance of the elderly to opioid therapy [7,92].

**Figure 3:**
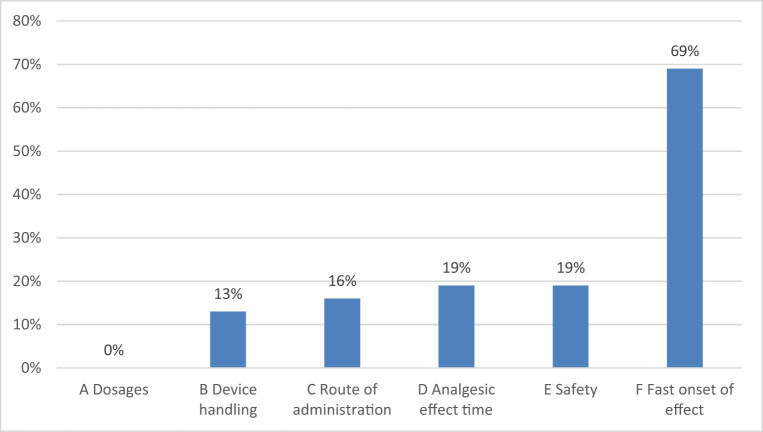
Survey question: “Which characteristic of a rapid onset opioid is the most important feature of your choice?.”

In our survey many experts choose BTcP treatment based on fast analgesic action to provide rapid pain relief, making “fast-onset effect” the main choice (69%) among multiple answers; the safety (19%) and duration of the analgesic effect (19%), which limit the administration of the next dose of opioids, are also important characteristics (Figure 3).

Ninety-one percent among 32 voters who responded to question 6 also believe it is an advantage to use the same molecule (fentanyl) to control basic pain and episodes of BTcP for easier management of opioid side effects; sixty-nine percent believe it was an advantage to have the availability of different dosages of ROO in order to adapt the therapy to the patient’s characteristics and type of pain.

Finally, all participants agreed that a multi-professional and multidisciplinary approach is necessary for the assessment and early treatment of cancer pain. Uncontrolled or undertreated pain may become physically and emotionally disabling, leading to increased suffering and reduced quality of life in every person living with a diagnosis of cancer, whether receiving treatment or not [93,94] and people consider unrelieved pain as an important factor affecting dignity at the end of life [95,96].

Diagnosing BTcP depends on the presence of well-controlled background pain, adequate control of baseline pain is an essential prerequisite to begin specific treatment for BTcP.

All clinicians including specialists and primary care physicians, who interact with cancer patients, will require the knowledge and the skills to implement best practices in the management of chronic pain and the education is one of the most important routes to enhance a new integration [97,98]. Nurses also need better training about chronic cancer pain and BTcP assessment and management, specifically to increase their ability to distinguish between breakthrough and background pain [99]. In the management of cancer pain, especially in the advanced phase of the disease and at the end of life, an additional opportunity will be provided by innovative e-health solutions, such as targeted Apps directly managed by the patient and telemedicine.

## 4 Conclusion

The availability of new cancer-directed treatments for advanced cancer reshapes cancer into a chronic illness and points to a basic challenge in cancer care. In every stage of illness, pain profoundly impacts individual functional status and quality of life, extending to families and community.

Many efforts have been made in Italy by Law 38/2010 to guarantee pain relief for citizens and to regulate opioid prescribing. New technologies provide drug formulations that respond to the need for personalised therapies and ensure efficacy and safety for the patient; however, resistance to opioid use remains, and low adherence to guidelines and recommendations on cancer pain management persists.

A disease-oriented approach to treatment is still prevalent, instead of an overall assessment, global, tailored care, and palliative care are often late and ineffective. Temel et al. [100] demonstrated that early provision of palliative care is associated with better quality of life, optimisation of symptom’s control, fewer depressive symptoms, less aggressive interventions at the end of life, improved longitudinal prognostic awareness, and longer survival.

Oncologists have more opportunities to make an early diagnosis of BTcP, as they see patients early and more often through the course of disease. Palliativists need to be aware of the high incidence of cancer and BTcP pain in their setting and pain therapists can offer valid invasive approaches in refractory pain, in selected cases. An effective integration of disciplines and close cooperation between oncologists, surgeons, pathologists, radiologists, palliativists, and pain specialists should increase a multidisciplinary psycho-socio-pharmacological approach in every setting and stage of disease trajectory, irrespective of whether treatment intention is curative, life-prolonging, or palliative. We hope that the Italian health care system will enhance well-planned, patient-centred care pathways for people with cancer in order to strengthen and ensure the practice of supportive and/or early palliative care for better symptom-management strategies and novel communication techniques. Similarly, changing the paradigm of referral palliative care to the end of life would ensure a more dignified path for the patients in advanced stage of illness.

Modern technologies can also strive to offer new drug delivery systems making opioid administration effective and safe for patients with cancer pain who need it.
